# The *Retrovirology *Open Access experience

**DOI:** 10.1186/1742-4690-6-115

**Published:** 2009-12-15

**Authors:** Kuan-Teh Jeang

**Affiliations:** 1 The National Institutes of Health, Bethesda, MD, USA

## Abstract

The *Retrovirology *Open Access experience after publishing more than 500 articles is discussed.

## Editorial

As 2009 comes to a close, it is instructive to reflect upon *Retrovirology's *experience with Open Access publishing. The journal was started with two objectives. First, there was a recognition that the robust field of basic retrovirus research could benefit from a dedicated rapid-publication online journal of good quality. Second, there was a desire to build a journal that would be freely accessible in full text to all readers without being restricted by the ability to pay for a subscription.

**Figure 1:**
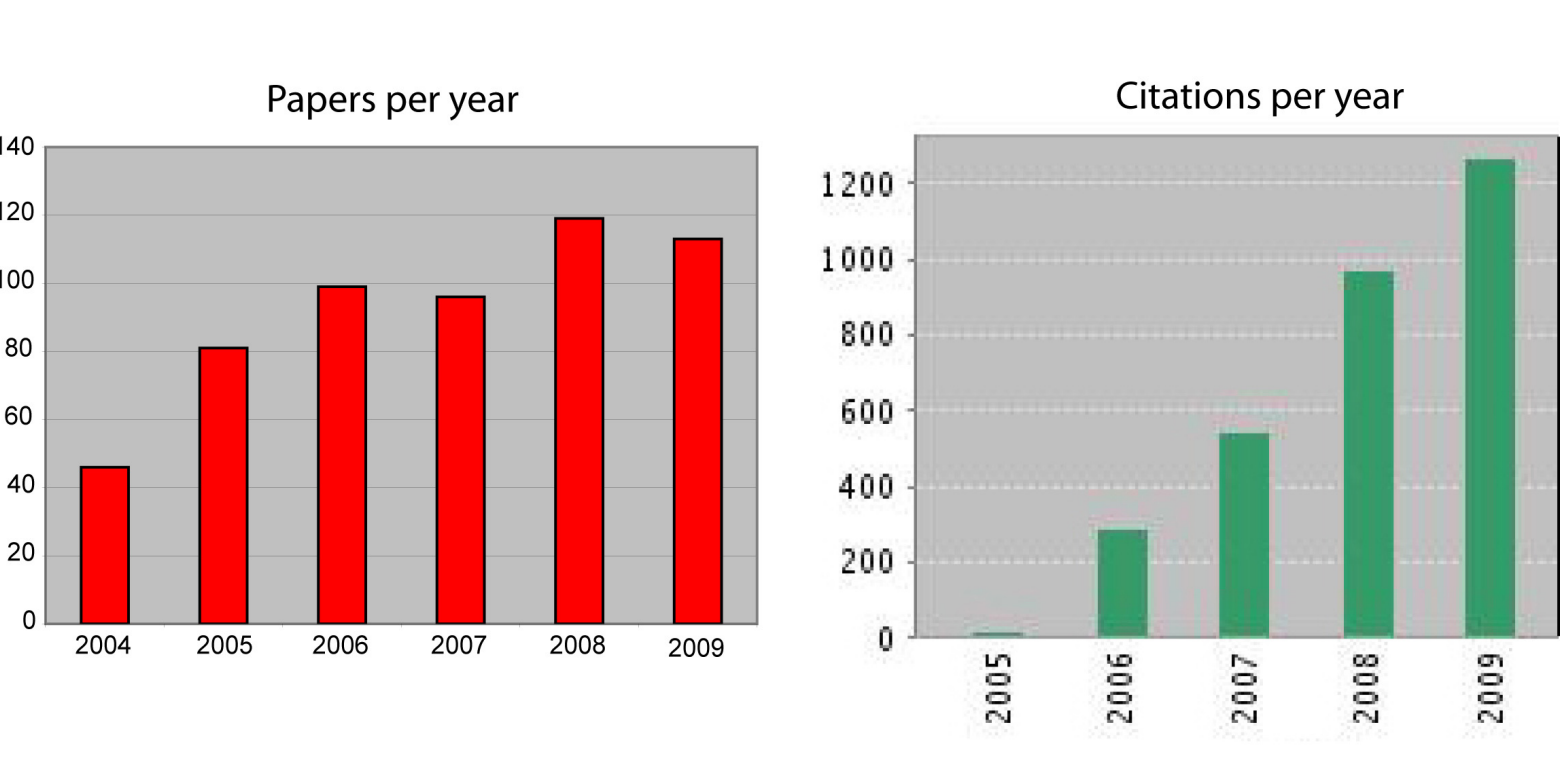
**Graphic representations of the number of published papers per year (left) and the number of citations to *Retrovirology *papers per year (right)**. Citation data are from the ISI Web of Science database. The numbers shown for 2009 are the information available at time of writing of this editorial and are not the final year-end numbers.

*Retrovirology *launched in February 2004 and since then has published more than 550 papers. To maintain a high scientific standard, the journal aims to have no more than 10 articles per month or roughly a total of 120 per year. From 2005 through 2009, *Retrovirology *has averaged ~100 published items per year (Figure 1). The quality of the journal has been monitored stringently by the editors and the editorial board and has improved over time. The latter assertion is supported by several observations. For instance, in November 2004, *Retrovirology *received 6 submissions and published 5 papers that month. By contrast, in November 2008, *Retrovirology *received 27 submissions and published 10 papers; and in November 2009, the journal received 29 submissions and published 10. In parallel, the rate of annual citations to *Retrovirology *has also increased steadily with a healthy upslope (Figure 1).

Open Access publishing in 2004 was viewed skeptically as a new approach with an uncertain future. In the beginning, many colleagues openly questioned whether an Open Access *Retrovirology *journal could be successful. Five years later, most subscription-based journals now offer their authors an Open Access option, and *Retrovirology*, as measured by SCImago journal rating http://www.scimagojr.com/ using data from Scopus, ranks in the top quartile of all virology journals. Similarly in data from the Journal Citation Reports of the ISI http://pcs.isiknowledge.com, *Retrovirology *has a recent Impact Factor [1] which is closely behind that of the *Journal of Virology*, and ahead of *Virology*, the *Journal of General Virology*, and *AIDS Research and Human Retroviruses*. The visibility of *Retrovirology *papers is attested by the citation numbers to recently published papers. For example, two *Retrovirology *review articles [2,3] published in 2007 and 2008 have already been cited 54 and 33 times, while two 2007 research papers [4,5] have been cited 27 and 23 times. These numbers are competitive with the citation frequencies to articles of similar age and similar topics published in other highly rated journals.

Periodically, emails arrive to me from colleagues in South America and graduate students in Africa conveying thanks for *Retrovirology's *fee-free full text Open Access format. As the journal's editor-in-chief, I am gratified by these responses. Perhaps on occasions when you are delayed in an airport lounge and need to read the full text of retrovirology papers using your personal lap top computer, you might be similarly gratified that *Retrovirology *is Open Access. The *Retrovirology *Open Access experience has been good for science, good for authors, and good for readers. The journal is doing well by doing good.

## Competing interests

The author is editor-in-chief of *Retrovirology*.

## Authors' contributions

KTJ wrote this editorial.
